# Use of piezoelectric surgery and Er:YAG laser:which one is more effective during impacted third molar surgery?

**DOI:** 10.1186/s40902-019-0212-6

**Published:** 2019-08-06

**Authors:** Seied Omid Keyhan, Hamid Reza Fallahi, Behzad Cheshmi, Sajad Mokhtari, Dana Zandian, Parisa Yousefi

**Affiliations:** 1 Stem cell & Regenerative Medicine Network, Shahid Beheshti University of Medical Sciences, Tehran, Iran; 2 School of Advanced Technologies in Medicine, Shahid Beheshti University of Medical Sciences, Tehran, Iran; 3 Dental Research Center, Research Institute of Dental Sciences, Shahid Beheshti University of Medical Sciences, Tehran, Iran; 4 Faculty of Dentistry, Boroujerd Islamic Azad University, Boroujerd, Iran; 5 Department of Prosthodontics, College of Dentistry, Isfahan University of Medical Sciences, Tehran, Iran

**Keywords:** Third molar, Wisdom tooth, Piezosurgery, Laser, Er:YAG

## Abstract

**Background:**

Reduction in postoperative complications is of vital considerations in impacted third molar teeth surgery. The aim of this study was to compare postoperative complications of impacted third molar surgeries for bone removal using laser, piezoelectric equipment, and conventional rotary instruments.

**Methods:**

To address the research purpose, the investigator designed the prospective double-blind clinical trial study. The sample size was determined 20 (40 teeth) by sampling formula in any kind of operation. The data of patients were obtained in the different periods in terms of pain, trismus, swelling, ecchymosis, and patient’s satisfaction and then analyzed using SPSS 20 software via paired *t* test and Wilcoxon and McNemar’s tests.

**Results:**

The pain immediately after surgery and 2 days and 7 days after surgery was higher in the laser group. The swelling immediately after surgery was more in the laser group but not significant. The amount of mouth opening immediately after surgery and 2 days and 7 days after surgery was significantly lower in the laser group than in the piezosurgery group. The total duration of surgery and duration of osteotomy were significantly longer in the laser group. The patient’s satisfaction from surgery with piezosurgery was more than that with laser, but this difference was not significant.

**Conclusion:**

Due to the rising demand for impacted wisdom tooth surgery, the present study suggests that hard tissue laser surgery and piezosurgery can clear the future of impacted molar surgery, and these approaches are more efficient in reducing postoperative complications compared to the conventional surgeries.

## Background

The impacted third molar teeth surgery is one of the most common operations in dentistry [1]. Studies conducted in the USA represent the exorbitant cost of removal of impacted third molars [2]. This sort of surgery can be simply performed in some patients using forceps and elevator; on the other hand, surgical intervention appears to be necessary in other patient groups, such as lifting the flap and removing the bone tissue [3]. The disadvantages of surgical complications should be assessed versus the benefits of teeth extraction [4].

The benefits of impacted third molar surgery include pain relief, prevention of caries and periodontal diseases, facilitation of orthodontic treatments and orthognathic surgeries, and prevention of pathological events such as the formation of dentigerous cysts and external root resorption in the adjacent teeth [2, 4].

Impacted third molar surgery similar to other surgical procedures is associated with intra- and post-operative complications [5]. Pain [6], swelling [7], ecchymosis [8], and trismus [9] are of the most common complications after third molar teeth extraction. The major concern in this type of surgery is the risk of damage to nerve [10], especially to the inferior alveolar nerve and lingual nerve during impacted mandibular third molar surgery, which can lead to numbness in the chin, lower lip, or tongue [11, 12].

The philosophy of the development of bone surgery by piezoelectric technique is based on two fundamental concepts in bone surgery: the minimum invasiveness and predictability of the surgery. Ease of control of the device can reduce bleeding during the surgery, and accurate cutting and excellent tissue healing are promising in the optimistic results of surgery, even in some cases with anatomical complexity [13].

In operations performed with piezosurgery, there is no need to apply extra force to overcome the reverse force caused by micro-motor rotation, and the force required for cutting is much lower; in addition to keeping the same depth of the cut, it also provides a better control for the surgeon and exerts less trauma to the mineralized tissues using the principles of biomechanics, as well as prevents excessive heat. It also causes minimal tissue damage to the bone by maintaining the life of osteocyte cells, resulting in reduced swelling and pain after surgery and experiencing shorter treatment course by the patient [14].

During the past two decades, lasers have been widely used in many branches of medicine. Initially, CO_2_ lasers were used for cutting mineral tissues [15, 16]. Erbium-doped yttrium aluminum garnet (Er:YAG) lasers are solid-state lasers that emit light with a wavelength of 2940 nm. Due to its wavelength that is precisely fit with the optical absorption spectrum of water and also is absorbed by the hydroxyapatite, these lasers are an efficient device in cutting rigid structures like bone, so that after the cutting, they leave only a superficial layer of bone with a size of a few micrometers [17, 18].

Several studies have attempted to compare bone removal techniques [19–21]. Some of these researches have reported very promising results related to laser surgery [19, 22]. Numerous studies have assessed several consequences including pain, swelling, trismus, ecchymosis, and patient’s satisfaction from the treatment [19, 23, 24]. The different outcomes of the studies have had many differences, and comparison of the laser with the piezosurgery in the few studies has not reported significant results in some cases [25–27].

The implementation of such a study to compare the postoperative complications of impacted teeth surgeries for bone removal between laser and piezoelectric equipment appears to be necessary. The purpose of the study was to evaluate post-operative complications after third molar surgery in laser-assisted and piezoelectric-assisted groups. The specific aim of the study was to compare post-operative complications between the two groups. The investigator hypothesized that no significant difference exists between the two groups.

## Material and methods

To address the research purpose, the investigator designed a prospective double-blind clinical trial study. The sample size was determined 20 (40 teeth) by sampling formula in any kind of operation. In order to perform the present study, the presence of bilateral impacted third molars was assessed in patients, and then inclusion and exclusion criteria were used for purifying the study group. The patients taking anticoagulants and immunosuppressants, those with systemic or local bone diseases, the patients undergoing radiotherapy, and pregnant women were excluded from the study. A random sample of 10 patients entered the case group, using piezosurgery in one side and Er:Yag laser on the other side to remove the mandibular wisdom teeth. Ten others entered the control group, using conventional rotary instruments to remove the mandibular wisdom teeth on both sides. Treatment design and subsequent complications were explained to the patients, and the patients signed written informed consent. This study has been designed and conducted in accordance with the recommendations of the Declaration of Helsinki for investigations with human subjects and has been approved by the Research Ethics Committee of Shahid Sadoughi University of Medical Sciences and Health Services under process number IR.SSU.REC.1395.184.

According to Pell and Gregory classification [29], the selected patients had similar type and impaction class on both sides of the mandible and the identical thickness of the bone that must be removed on both sides in the panoramic image. The patients underwent surgery using appropriate anesthesia and according to routine treatment process by a single oral and maxillofacial surgery residency.

The patient’s teeth were randomly operated by piezosurgery on one side and the laser on the other side. For this purpose, a list of the patients’ names was provided, and the surgery side for laser and piezo equipment was randomly determined via the computer.

The hockey-stick surgical incision was performed on the buccal side, and lingual flap was removed to protect the lingual nerve. In both groups, either piezo or laser was used for osteotomy (bone removal), and if there was a need for teeth section, dental bur was used on both sides in order to avoid interference.

The laser used in this study was the Er:YAG Fidelis plus III laser (Fotona Co., Slovenia). In the teeth group operated by laser, the surgeon used protective glasses and the same condition of the laser with a wavelength of 2.94 μm and power of 20 W that was set to the duration of each pulse of 100 μs, energy of each pulse of 350 mJ, and frequency of 20 Hz. The handpiece used for the laser was non-contact in which the distance between the laser tip and the bone surface was 10 mm. In all the samples, a gauge was used to unify distance between the laser tip and the bone surface.

**Fig. 1:**
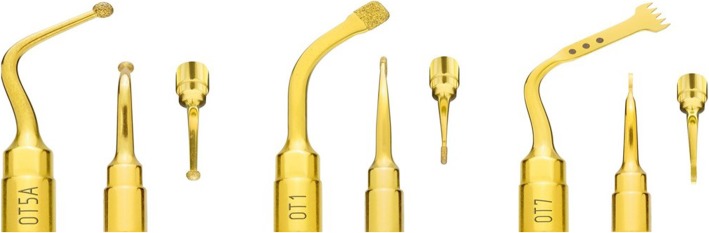
The piezosurgery tips used in this study are OT1, OT7, and OT5A

The piezoelectric surgery device applied in this study was Piezosurgery 3 (Mectron Co.), which all parts were sterilized after each use. New scaler tips special for osteotomy (OT1, OT7, OT5A) were used for the bone incision (Fig. 1).

**Fig. 2:**
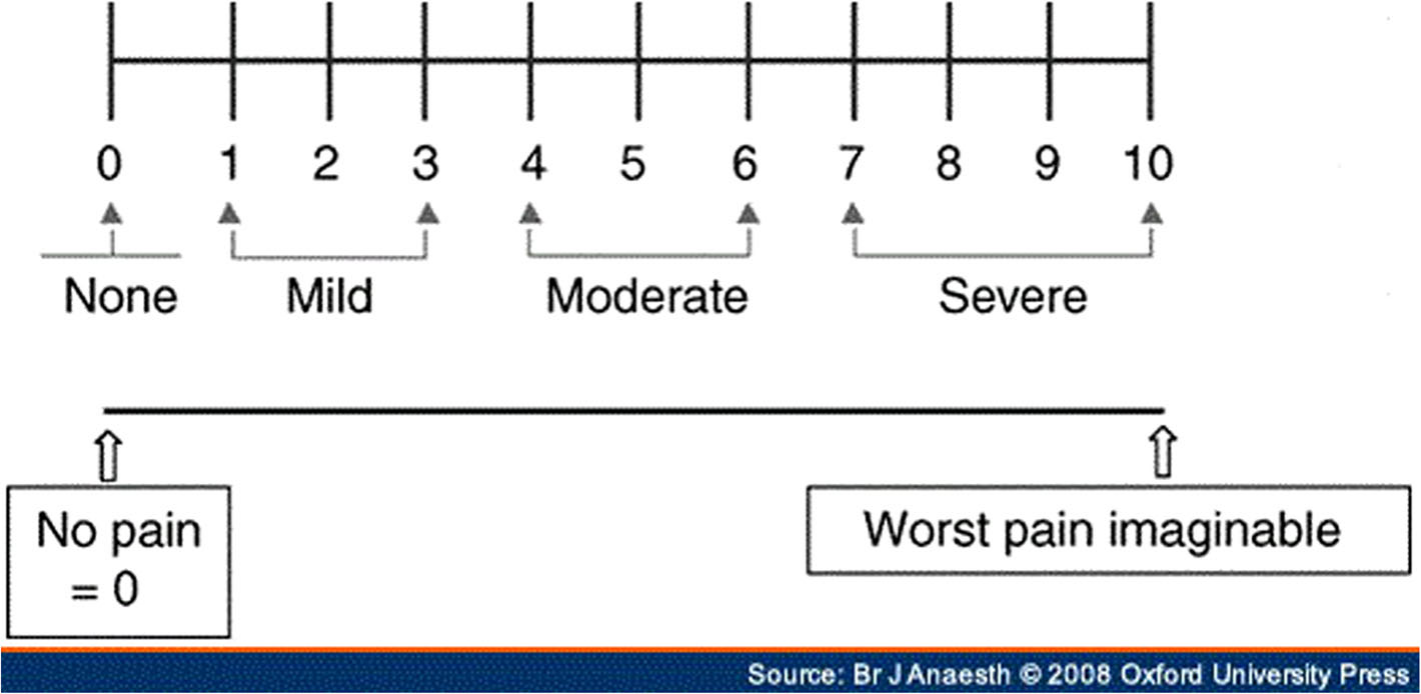
Visual analog scale

**Fig. 3:**
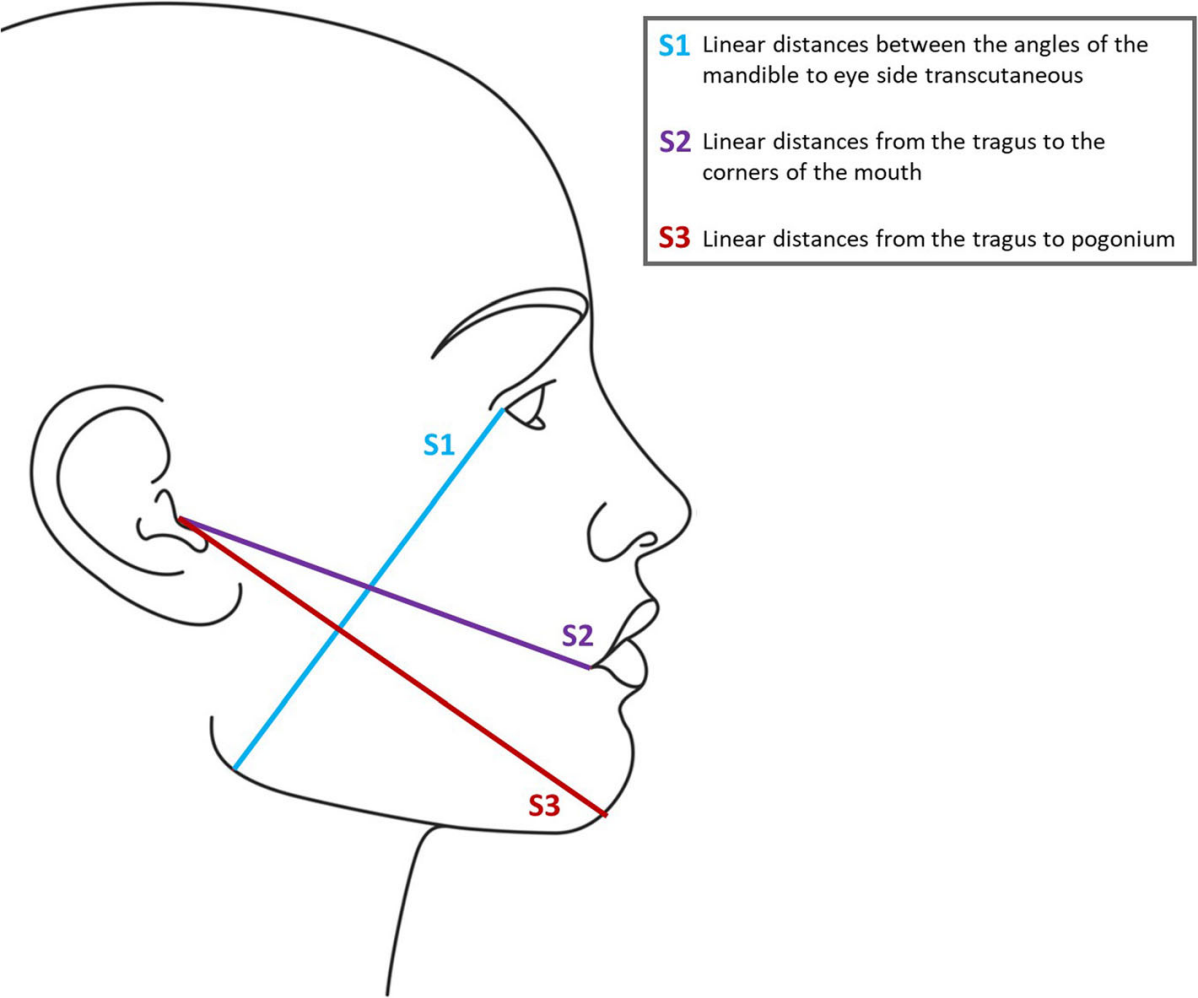
Assessing swelling level by Gabka and Matsumara technique

After surgery, routine rinsing was performed with normal saline, the flap was returned to its previous anatomic status, and then suturing was done with silk suture thread. The patients were assessed in terms of respective factors for postoperative problems before surgery, immediately after surgery, and 2 and 7 days after surgery. Visual analog scale (VAS) was used for pain measurement from 1 to 10 (Fig. 2). In order to measure the trismus, the distance between the upper and lower right incisors was measured at the maximum amount of mouth opening. To assess swelling level by Gabka and Matsumara technique [30], linear distances between the angles of the mandible to eye side transcutaneous (S1), from the tragus to the corners of the mouth (S2), and from the tragus to pogonium (S3) were measured by a caliper with sensitivity of 0.02 mm (Fig. 3). The sum of these sizes was calculated as the facial dimension and used to measure the swelling level. Clinical images were taken at specified times to investigate the level of ecchymosis. Three independent controls, who were unaware of the type of surgery on each side, observed the images and graded the ecchymosis level between 0 and 3, because there is no standard grading scale for ecchymosis (0 = no ecchymosis, 1 = bruise in the angle of the mandible, 2 = bruise on the angle extending to the mandibular border and buccal region, 3 = bruise beyond the mandibular border). On the seventh day after surgery, patient’s satisfaction from the treatment was asked from 0 to 4 in each side (0 = very poor, 1 = poor, 2 = average, 3 = good, 4 = excellent).

Duration of surgery in the three intervals including *T*_1_ = from the injection of the anesthetic drug until completely removing the flap, *T*_2_ = duration of bone removal and wisdom teeth extraction, and *T*_3_ = duration of the rinsing cavity and suturing was recorded by residents.

Blinding was performed to measure each factor, and the clinician was unaware of the type of treatment in each side of the patient.

Data were noted in the checklist and then were inserted to SPSS 20 software for analysis. After collecting data, they were encoded and inserted into the computer and then analyzed using SPSS 20 software via paired *t* test and Wilcoxon and McNemar’s tests.

## Results

Our results demonstrated that the pain immediately after surgery and 2 days and 7 days after surgery is higher in the laser group insignificantly (Table 1). In addition, the swelling immediately after surgery is slightly higher in the laser group but not significant. Swelling at 2 days after surgery is significantly higher in the piezosurgery group but insignificantly higher at 7 days after surgery (Table 2). The trismus immediately after surgery and 2 days and 7 days after surgery was significantly lower in the laser group (Table 3). The results of investigating ecchymosis presented the lack of ecchymosis in all groups. Moreover, the total duration of surgery and duration of osteotomy were significantly longer in the laser group. The patient’s satisfaction from surgery with piezosurgery was more than that with laser surgery, but this difference was not significant (Table 4).

**Table 1:** Comparison of pain variable in the three surgical techniques

Paired t test (*P* value)	Conventional rotary instrument		Laser		Piezosurgery		Pain
	S.D	Mean	S.D	Mean	S.D	Mean	
	0	0	0	0	0	0	Before surgery
0.055	35.1	25.6	1.447	6.33	1.113	5.67	After surgery
0.531	37.2	5	1.352	3.40	1.082	3.20	Day 2 after surgery
0.546	99.2	41.3	0.704	1.07	0.704	0.93	Day 7 after surgery

**Table 2:** Comparison of facial dimension variable to investigate the swelling in the three surgical techniques

Paired *t* test (*P* value)	Conventional rotary instrument		Laser		Piezosurgery		Swelling
	S.D	Mean	S.D	Mean	S.D	Mean	
0.647	43.0	22.386	1.905	38.662	1.892	38.657	Before surgery
0.117	33.0	38.993	1.99	988/38	1.879	38.852	After surgery
0.0001	34.0	61.398	2.022	39.80	1.884	40.75	Day 2 after surgery
0.128	39.0	38.829	1.939	38.899	1.924	38.991	Day 7 after surgery

**Table 3:** Comparison of the amount of mouth opening to investigate trismus in the three surgical techniques

Paired *t* test (*P* value)	Conventional rotary instrument		Laser		Piezosurgery		Trismus
	S.D	Mean	S.D	Mean	S.D	Mean	
	48.0	88.4	0.599	4.69	0.599	4.69	Before surgery
0.025	69.0	19.3	0.739	4.227	0.635	4.367	After surgery
0.0001	70.0	70.3	0.606	3.027	0.685	3.59	Day 2 after surgery
0.004	80.0	30.4	0.742	4.153	0.772	4.38	Day 7 after surgery

**Table 4:** Comparison of surgery duration and patient’s satisfaction in the two surgical techniques

Paired *t* test (*P* value)	Laser		Piezosurgery		Variable
	S.D	Mean	S.D	Mean	
0.0001	3.081	9.93	2.165	6.60	Osteotomy duration (t2)
0.0001	3.464	27	3.109	22.33	Total surgery duration (tt)
0.096	0.594	2.73	0.594	3.07	Patient’s satisfaction

The pain immediately after surgery and 2 days and 7 days after surgery was significantly higher than before the operation. The pain at 2 and 7 days after surgery was significantly decreased compared to that immediately after surgery.

The pain in the piezosurgery group was significantly decreased 7 days after surgery compared to 2 days after surgery; the swelling immediately after surgery and at 2 and 7 days after surgery was significantly higher than that before surgery; the swelling at 2 and 7 days after surgery was significantly increased compared to that immediately after surgery; and the swelling at 7 days after surgery was significantly decreased than that at 2 days after surgery (Table 5).

**Table 5:** Comparison of pain and swelling variables at different times of piezoelectric surgery

Paired *t* test (*P* value)	Piezosurgery (t2)		Piezosurgery (t1)		Variables
	S.D	Mean	S.D	Mean	
0.0001	1.113	5.67	0	0	Pain before surgery (t1) and after surgery (t2)
0.0001	1.082	3.20	0	0	Pain before surgery (t1) and day 2 after surgery (t2)
0.0001	0.704	0.93	0	0	Pain before surgery (t1) and day 7 after surgery (t2)
0.0001	1.082	3.20	1.113	5.67	Pain after surgery (t1) and day 2 after surgery (t2)
0.0001	0.704	0.93	1.113	5.67	Pain after surgery (t1) and day 7 after surgery (t2)
0.0001	0.704	0.93	1.082	3.20	Pain day 2 after surgery (t1) and day 7 after surgery (t2)
0.0001	1.879	38.852	1.892	38.657	Swelling before surgery (t1) and after surgery (t2)
0.0001	1.884	40.750	1.892	38.657	Swelling before surgery (t1) and day 2 after surgery (t2)
0.0001	1.924	38.991	1.892	38.657	Swelling before surgery (t1) and day 7 after surgery (t2)
0.0001	1.884	40.75	1.879	38.852	Swelling after surgery (t1) and day 2 after surgery (t2)
0.009	1.924	38.991	1.879	38.852	Swelling after surgery (t1) and day 7 after surgery (t2)
0.0001	1.924	38.991	1.884	40.75	Swelling day 2 after surgery (t1) and day 7 after surgery (t2)

In the piezosurgery group, the amount of mouth opening immediately after surgery and 2 days and 7 days after surgery was significantly decreased compared to that before surgery. In addition, the amount of mouth opening at 2 days after surgery was significantly decreased than that immediately after surgery; the amount of mouth opening at 7 days after surgery was greater than that immediately after surgery, but not significantly; and the amount of mouth opening at 7 days after surgery was significantly more than that at 2 days after surgery (Table 6).

**Table 6:** Comparison of trismus variable at different times of piezoelectric surgery

Paired *t* test (*P* value)	Piezosurgery (t2)		Piezosurgery (t1)		Variables
	S.D	Mean	S.D	Mean	
0.0001	0.635	4.367	0.599	4.693	Trismus before surgery (t1) and after surgery (t2)
0.0001	0.685	3.59	0.599	4.693	Trismus before surgery (t1) and day 2 after surgery (t2)
0.016	0.772	4.38	0.599	4.693	Trismus before surgery (t1) and day 7 after surgery (t2)
0.0001	0.685	3.59	0.635	4.367	Trismus after surgery (t1) and day 2 after surgery (t2)
0.898	0.772	4.38	0.635	4.367	Trismus after surgery (t1) and day 7 after surgery (t2)
0.0001	0.772	4.38	0.685	3.59	Trismus day 2 after surgery (t1) and day 7 after surgery (t2)

In the laser group, the pain immediately after surgery and 2 days and 7 days after surgery was significantly higher than that before surgery; the pain at 2 and 7 days after surgery was significantly decreased compared to that immediately after surgery; and the pain at 7 days after surgery was significantly decreased compared to that at 2 days after surgery.

The swelling immediately after surgery and 2 days and 7 days after surgery was significantly higher than that before surgery; the swelling at 2 days after surgery was significantly increased compared to that after surgery. The swelling at 7 days after surgery was reduced compared to that immediately after surgery, but not significantly; the swelling at 7 days after surgery was significantly decreased compared to that at 2 days after surgery (Table 7).

**Table 7:** Comparison of pain and swelling variables at different times of laser surgery

Paired *t* test (*P* value)	Laser (t2)		Laser (t1)		Variables
	S.D	Mean	S.D	Mean	
0.0001	1.447	6.33	0	0	Pain before surgery (t1) and after surgery (t2)
0.0001	1.352	3.40	0	0	Pain before surgery (t1) and day 2 after surgery (t2)
0.0001	0.704	1.07	0	0	Pain before surgery (t1) and day 7 after surgery (t2)
0.0001	1.352	3.40	1.447	6.33	Pain after surgery (t1) and day 2 after surgery (t2)
0.0001	0.704	1.07	1.447	6.33	Pain after surgery (t1) and day 7 after surgery (t2)
0.0001	0.704	1.07	1.352	3.40	Pain day 2 after surgery (t1) and day 7 after surgery (t2)
0.0001	1.925	39	1.905	38.662	Swelling before surgery (t1) and after surgery (t2)
0.0001	2.022	39.8	1.905	38.662	Swelling before surgery (t1) and day 2 after surgery (t2)
0.002	1.939	38.899	1.905	38.662	Swelling before surgery (t1) and day 7 after surgery (t2)
0.0001	2.022	39.8	1.925	39	Swelling after surgery (t1) and day 2 after surgery (t2)
0.195	1.939	38.899	1.925	39	Swelling after surgery (t1) and day 7 after surgery (t2)
0.0001	1.939	38.899	2.022	39.8	Swelling day 2 after surgery (t1) and day 7 after surgery (t2)

In the laser group, the amount of mouth opening immediately after surgery and 2 days and 7 days after surgery was significantly decreased compared to that before surgery; the amount of mouth opening at 2 days after surgery was significantly decreased compared to that immediately after surgery; and the amount of mouth opening in the laser group at 7 days after surgery was less than that immediately after surgery insignificantly. The amount of mouth opening in the laser group at 7 days after surgery was significantly increased compared to that at 2 days after surgery (Table 8).

**Table 8:** Comparison of trismus variable at different times of laser surgery

Paired *t* test (*P* value)	Laser (t2)		Laser (t1)		Variables
	S.D	Mean	S.D	Mean	
0.0001	0.739	4.22	0.599	4.69	Trismus before surgery (t1) and after surgery (t2)
0.0001	0.606	3.02	0.599	4.69	Trismus before surgery (t1) and day 2 after surgery (t2)
0.001	0.742	4.15	0.599	4.69	Trismus before surgery (t1) and day 7 after surgery (t2)
0.0001	0.606	3.02	0.739	4.22	Trismus after surgery (t1) and day 2 after surgery (t2)
0.43	0.742	4.15	0.739	4.22	Trismus after surgery (t1) and day 7 after surgery (t2)
0.0001	0.742	4.15	0.606	3.02	Trismus day 2 after surgery (t1) and day 7 after surgery (t2)

In the control group, the pain immediately after surgery and 2 days and 7 days after surgery was significantly higher than that before surgery. The pain at 2 and 7 days after surgery was significantly decreased compared to that immediately after surgery. The pain at 7 days after surgery was significantly decreased compared to that at 2 days after surgery; the swelling immediately after surgery and 2 days and 7 days after surgery was higher than that before surgery; and the swelling at 2 days after surgery was increased compared to that after surgery, but not significantly. The swelling at 7 days after surgery was reduced compared to that immediately after surgery; the swelling at 7 days after surgery was significantly decreased compared to that at 2 days after surgery. Differences for the swelling criteria in the control group were not statistically significant.

In the control group, the amount of mouth opening immediately after surgery and 2 days after surgery was decreased compared to that before surgery. The amount of mouth opening at 2 days after surgery was significantly increased compared to that immediately after surgery; the amount of mouth opening in the control group at 7 days after surgery was significantly more than that immediately after surgery. The amount of mouth opening at 7 days after surgery was significantly increased compared to that at 2 days after surgery. Control group data are collected in Tables 1, 2 and 3.

## Discussion and conclusion

In order to overcome the limitations of old-style ultrasonic surgery in which the conventional piezoelectric equipment was used, Tomaso Vercellotti et al. began to develop ideal ultrasonic technology for incision of the bone. The results of the experimental phase of laboratory investigations on animals’ bone created a primary prototype, called piezosurgery [28]. In other studies [29], the therapeutic effects of laser procedures (Er:YAG) were investigated, which affected the old-style ultrasonic methods.

According to the present results, the pain immediately after surgery and 2 days and 7 days after surgery was higher in the laser group that there was no significant difference in any periods. Also, the swelling immediately after surgery and 2 days after surgery was significantly higher in the laser group, but the swelling at 7 days after surgery in the piezosurgery group was higher; there was no significant difference. The amount of mouth opening immediately after surgery and 2 days and 7 days after surgery was significantly lower in the laser group than in the piezosurgery group, indicating significantly higher trismus in the laser group. Moreover, the total duration of surgery and duration of osteotomy were significantly longer in the laser group.

Rud [20] in a prospective study investigated the impacted mandibular third molar surgery in 52 patients using conventional surgery via drilling (group A) and surgery by the piezoelectric device (group B) using the Parant scale with simple and complex categories. They reported that when complex extraction of the mandibular third molar was carried out, assessment of pain and surgery duration was recorded shorter, and when the extraction was simple, the duration of surgery was similar in both groups. Nevertheless, the pain in the first day of surgery procedure by drilling was much higher than that of other methods. Osteonecrosis of the bone was observed only in the rotational group and a high level of alkaline phosphatase enzyme in the piezoelectric group. Finally, it was reported that in the longer interventions, the pain after extraction and third molar surgery problems would be much more.

Sortino et al. [21] conducted a study on 100 patients with the mandibular third molar problem, in which 50 patients using rotational osteotomy technique (group A) and 50 patients using piezoelectric osteotomy technique (group B) were treated. The treatment protocol was the same, and the facial swelling and trismus were examined 24 h after surgery. They reported that the average duration of surgery was 17 min in group A and 23 min in group B. The swelling level in the rotational osteotomy group (7.04 mm vs. 4.22 mm) and trismus (16.7 mm vs. 12.5 mm) were much more than in the piezoelectric group. Postoperative trismus and facial swelling in the piezoelectric osteotomy group (group B) showed a significant decrease while the longer surgery duration is required. Finally, it was reported that the piezoelectric osteotomy technique (group B) within 24 h after surgery was very effective in reduction of swelling and trismus, in line with our results.

In the study of Basheer et al. [30], 30 adults requiring treatment of third molars were divided into two groups of 15. The first group was treated with the piezoelectric osteotomy technique and the second group with the rotational osteotomy technique. The rotary dental instrument was with a rotational speed of 35,000 rpm, and the piezoelectric instrument was with the frequencies of 25 to 29 kHz with microvibration of 60 to 200 mm/s. The mean age of the patients in the piezosurgery group was 28 years and in the rotational group was 30 years. The results reported that the surgery duration in the rotational technique was shorter than that in the piezoelectric technique and the pain intensity in the rotational group was higher up to 4 days after surgery. However, the amount of mouth opening in the piezoelectric group was significantly better than that in the rotational group (rotary dental bur) up to 7 days after surgery. Finally, it was reported that on the piezosurgery day, postoperative pain, trismus, and swelling were reduced and may also play an important role in increasing the bone density in the extraction cavity and reducing bone loss from adjacent teeth in the distal area, which is similar to the obtained results of the present study.

Al-Moraissi et al. [31] carried out a systematic review and meta-analysis aiming to respond to the question of whether or not the piezoelectric surgical procedure has less postoperative complications in third molar surgery compared to the common rotational surgical procedure. They reported that a significant difference was found between piezoelectric surgery and rotational surgery regarding the postoperative complications including edema, trismus, and pain, as well as the total number of sedative consumed to reduce the pain. They showed that piezosurgery technique significantly reduces these complications. However, the increase in the duration of surgery was very clear and significant in the piezosurgery group.

The study of Jiang et al. [32] investigating possible complications of piezosurgery and conventional rotary instrument technique in third molar surgery in a clinical trial showed that the duration of surgery in the piezosurgery technique was higher than that in the other techniques, including rotary instruments (4.13 min). As well, the postoperative pain, swelling, and trismus in the piezosurgery group at 1, 3, 5, and 7 days were significantly lower. Finally, it was reported that the piezosurgery technique is a promising alternative technique to extract impacted molar.

In the study of Bartuli et al. [33] conducted on the surgical procedures for impacted third molar with high speed by piezosurgery technique and handpiece, 192 patients were selected and studied surgical techniques were randomly applied on the subjects. Analgesic treatment with 1000-mg paracetamol tablets and postoperative pain questionnaire (Wong-Baker FACES pain rating scale questionnaire) were used. Finally, it was found that the mean duration in the handpiece technique was much lower than that in the piezosurgery technique (34 min vs. 54 min), while the pain level was the same for both techniques. As a result, it can be concluded that osteotomy by traditional techniques can still be a golden standard in impacted molar surgery; piezosurgery can be considered particularly to maintain the anatomical structure of the bone.

Pippi and Alvaro [34] reported in their study that piezosurgery can be a highly effective technique for the removal of third molars. Its only weakness is the long surgery duration, which is considered lower than the piezoelectric instrument due to the power outages; such a result was also obtained in the study of Abu-Serriah et al. [22], and it warns about the longer duration of surgery in patients undergoing laser surgery.

Romeo et al. [35] indicated that third molar surgery by YAG laser can significantly decrease pain, trismus, and swelling compared to the rotational osteotomy; also, the longer surgery duration in the rotational osteotomy group was highly significant, which indicates that laser can be used as an alternative technique to conventional rotational surgery.

In the study of Passi et al. [24], it was reported that the amount of pain, hemorrhage, and swelling in the laser group was less than that dental bur and rotational surgery group, but the passed time in the laser group was double compared to the group of incision with dental rub. Also, Romeo et al. [36] stated faster healing and the incidence of thermal damage in the laser-test group.

Based on the reviewed researches, the piezosurgery technique can be a promising method to the surgical treatment of impacted third molars. However, its long duration of surgery has been investigated in these studies, but studies indicate that the piezosurgery method can be more effective to reduce the pain, trauma, and trismus compared to the conventional surgical techniques (dental burs or rotary). Concerning laser surgery, it can be concluded that the studies indicate that laser surgery and piezosurgery will improve the future of third molar surgeries. The only problem of these surgeries for surgeons is its long duration, but since the purpose is to reduce the pain and complications in the patients, the long duration of these procedures can be justified.

Given the rising demand for impacted molar surgery, this study suggests that laser surgery and piezosurgery techniques can improve the future of the impacted molar surgery. These approaches are more efficient in reducing postoperative complications compared to the conventional surgeries. Although the duration of surgery can be increased in these operations, it can be justified by reducing these surgical complications.

## Data Availability

The datasets generated and/or analyzed during the current study are not publicly available due but are available from the corresponding author on reasonable request.

## Abbreviation

Er:YAG laser: Erbium-doped yttrium aluminum garnet laser
